# Ecological Editing: Decoupling Microbial Signals from Metabolic Noise to Reprogram the Gut Ecosystem

**DOI:** 10.3390/microorganisms14102410

**Published:** 2026-10-21

**Authors:** Yu He, Xiaojian Zhu, Zhijiang Huang, Xiaoyuan Yang, Bo Yi

**Affiliations:** 1 2nd Abdominal Surgery Department, Jiangxi Cancer Hospital, The Second Affiliated Hospital of Nanchang Medical College, Nanchang 330029, China; heyu@email.ncu.edu.cn (Y.H.); 410014230039@email.ncu.edu.cn (Z.H.); 15070058340@163.com (X.Y.); 2 Jiangxi Medical College, Nanchang University, Nanchang 330006, China; 3 Jiangxi Key Laboratory of Oncology, Jiangxi Cancer Hospital, Nanchang 330006, China; 4 Jiangxi Province Key Laboratory of Drug Target Discovery and Validation, School of Pharmacy, Jiangxi Medical College, Nanchang University, Nanchang 330031, China; 5 Jiangxi Provincial Key Clinical Specialty Construction Project—Colorectal Cancer Center, Jiangxi Cancer Hospital, The Second Affiliated Hospital of Nanchang Medical College, Nanchang 330029, China

**Keywords:** ecological editing, quantitative fractionated microbiota (QFM), washed microbiota transplantation (WMT), live biotherapeutic products (LBPs), soil-and-seed strategy, immunological sieve

## Abstract

The translation of microbiome therapeutics is hampered by the stochasticity of faecal microbiota transplantation (FMT), an empirical transfer of undefined biomass confounded by donor heterogeneity, procedural variability, and immunogenic debris. As the field moves towards precision medicine, microbiome engineering is diverging into two philosophies: the subtractive paradigm, which purifies the native ecosystem by removing deleterious metabolites, and the additive paradigm, which assembles functional guilds de novo. In this review, we propose that density-based ecological purification, termed quantitative fractionated microbiota (QFM), may act as an immunological sieve, using washed microbiota transplantation (WMT) as its best-characterised clinical exemplar, to separate therapeutic signals (viable microbiota and bacteriophages) from metabolic noise (for example, pro-inflammatory lipid mediators and glucocorticoid metabolites). We explicitly frame this separation as a conceptual working model rather than an established mechanism: the quantitative assays, cut-off values, and units required to operationalise a signal-to-noise distinction do not yet exist, and we set out what would be needed to develop them. Conversely, live biotherapeutic products (LBPs) offer compositional precision but face a critical bottleneck, namely engraftment failure in colonisation-resistant hosts. We propose that the future lies less in competition between these modalities than in their convergence, which we term the soil-and-seed framework. In this framework, subtractive ecological priming (QFM/WMT) may transiently lower colonisation resistance by liberating niche space and by reducing soluble inflammatory exposure, thereby preparing the mucosa for subsequent engraftment of additive consortia. We describe this hybrid strategy as a hypothesis with an explicit validation roadmap. If it holds, this shift would move microbiome therapy from stochastic biomass transfer towards predictable ecosystem engineering.

## 1. Introduction

### 1.1. From Empirical Ecosystem Transfer to Rational Signal Extraction

For over a decade, faecal microbiota transplantation (FMT) has been viewed through a reductionist lens, as a form of transfusion for a missing microbial organ [1,2]. This analogy lacks pharmacological precision. Unlike sterile blood products with defined pharmacokinetics, FMT transfers an evolving ecosystem containing thousands of biotic agents together with a large reservoir of abiotic metabolites and host-derived material [3,4,5]. FMT achieves high cure rates in recurrent *Clostridioides difficile* infection (rCDI) [6,7,8], yet its translation to complex chronic pathologies—ulcerative colitis (UC) [9,10], metabolic-dysfunction-associated steatotic liver disease [11,12], and immunotherapy-refractory cancers [13,14]—has been inconsistent, and syntheses continue to report substantial heterogeneity between trials [15,16].

The heterogeneity of clinical outcomes, often described as donor-dependent efficacy or the “super-donor” phenomenon [17], suggests that success depends not only on which taxa are transferred but on what else accompanies them [16,18,19]. We therefore adopt two working definitions. Signal denotes the viable, engraftable biotic payload, principally bacteria and bacteriophages, together with the ecological interactions that sustain it. Noise denotes non-viable or soluble components of the donor matrix that can acutely engage host sensing pathways, including pro-inflammatory lipid mediators, damage-associated molecular patterns (DAMPs), free antigens, and glucocorticoid metabolites. Crude FMT delivers a potent biological signal but presents it within a complex, immunologically active matrix [3,20,21].

### 1.2. Literature Search Strategy

This review is a narrative synthesis rather than a systematic review, and no formal risk-of-bias assessment was performed. We searched PubMed/MEDLINE, EMBASE, and Web of Science from database inception to 31 January 2026, with a final update on 30 September 2026, combining controlled vocabulary and free-text terms across three concept blocks: (i) microbiota transfer (faecal/fecal microbiota transplantation, washed microbiota transplantation, sterile faecal filtrate transfer, microbiota-based therapy); (ii) manufacturing and regulation (fractionation, ethanol inactivation, spore purification, live biotherapeutic product, defined consortium, good manufacturing practice, potency assay, release criteria); and (iii) mechanism (colonisation resistance, engraftment, bile acid, short-chain fatty acid, leukotriene B4, prostaglandin E2, bacteriophage, mucosal immunity). We also hand-searched the reference lists of retrieved reviews, the registries ClinicalTrials.gov and ChiCTR, and the safety communications of the United States Food and Drug Administration (FDA) and the European Medicines Agency. Records were included if they reported primary clinical, microbiological, immunological, manufacturing, or regulatory data relevant to microbiota processing, or if they were consensus statements from professional societies. Conference abstracts, non-peer-reviewed preprints and studies reporting only unprocessed taxonomic associations without a therapeutic or manufacturing endpoint were excluded. Where evidence is derived from animal models, single-centre series, or a single research group, this is stated explicitly in the text.

### 1.3. Hypothesis and Scope

We propose a dual-process strategy, the soil-and-seed framework, that combines subtractive removal of deleterious noise through quantitative fractionation (exemplified by washed microbiota transplantation, WMT) with additive introduction of defined functional modules through live biotherapeutic products (LBPs) [22,23,24]. We use the term “ecological editing” for this combination. Two caveats frame the entire review. First, the signal-to-noise distinction is presented as a conceptual paradigm, not as a measurable ratio with defined units; no validated assay, threshold, or unit for it currently exists. Second, the mechanistic links we describe are hypotheses of differing evidential strength, and in Section 5, we classify each link according to whether it is supported by direct human data, extrapolated from model systems, or speculative. By preparing the ecological soil before planting the seed, we outline a research agenda for overcoming colonisation resistance and for achieving reproducible ecosystem reprogramming.

## 2. The Subtractive Paradigm: Engineering Principles of Purified Microbiota

### 2.1. From Crude Matter to Defined Agents: The Methodological Evolution

The preparation of faecal microbiota has undergone substantial evolution [25]. Early protocols relied on heterogeneous manual processing; modern stool banks have since established standards for automated homogenisation, donor screening, and cryopreservation, including the European consensus conference recommendations and the operating standards of large stool banks [26,27,28,29]. A notable epistemological divergence nonetheless persists. Most current international standards prioritise retention of the whole stool matrix in order to maximise biomass recovery, implicitly treating the co-transferred soluble environment as benign or beneficial [28,29]. Whether that assumption is justified has, to our knowledge, never been tested in a randomised design.

We delineate a counter-paradigm, quantitative fractionated microbiota (QFM). With WMT serving as the best-characterised clinical exemplar, this approach is not merely a sanitisation protocol but a distinct methodology of purification by differential sedimentation and size-based sieving [22,30,31]. QFM strategies exploit differences in sedimentation behaviour and particle size between microbial cells and host-derived matrix components. This distinction matters because, as we emphasise in Section 2.2, washed preparation is frequently mislabelled as density-gradient centrifugation. In principle, this physical segregation pursues three objectives:

Signal–noise decoupling: segregating the solid microbial phase from the soluble supernatant that carries inflammatory mediators.
Ligand depletion: reducing the concentration of soluble pro-inflammatory ligands such as leukotriene B4 (LTB4) and free antigens in the administered product [22]. We note that the threshold below which such ligands fail to activate mucosal immune sensors has not been defined quantitatively for any of these mediators in the transplant setting.
Payload quantification: converting the graft from a variable faecal suspension into a counted number of microbial particles, which permits dose reporting in units that are comparable between preparations.

From a manufacturing and regulatory perspective, however, washed preparation occupies an intermediate position on the microbiome-therapeutics continuum. Defined single-strain and synthetic consortia LBPs have fully sequenced identities, clonal master cell banks, and delineated purity profiles, whereas a washed preparation remains an undefined biological mixture derived from a human donor ecosystem [32,33]. Achieving genuine pharmaceuticalisation therefore requires progress beyond biomass quantification towards a chemistry, manufacturing, and controls (CMC) architecture, functionally qualified potency matrices, and defensible clinical release parameters, which we set out in Section 2.9.

### 2.2. Technological Standardisation: From Artisanal Filtration to Automated Fractionation

Distinct from manual filtration, WMT employs an automated, closed-loop preparation system grounded in quantitative fractionation [22,34]. The engineering logic rests on sedimentation kinetics combined with pore-size sieving: by subjecting the suspension to controlled, repeated low-speed centrifugal cycles and multi-stage microfiltration, the system sediments intact microbial biomass while retaining soluble small molecules, free endotoxin, and low-density debris in the supernatant, which is discarded. No chemical density gradient (for example, Percoll, sucrose, or iodixanol) is used, and the process should not be described as density-gradient centrifugation; early descriptions that used this term conflated two different unit operations [22,34]. This workflow redefines the preparation standard, moving from simple filtration towards purification of a defined biological payload and enabling quantitative quality control (QC) [22,34].

Numerical standardisation of physical bacterial units nevertheless addresses only mass consistency. In bioprocessing, biological activity does not scale linearly with absolute particle counts [33]. Genuine batch consistency requires pairing particle enumeration with functional viability assays, taxonomic integrity monitoring, and biological activity thresholds, so that variation arising from donor circadian, dietary, and seasonal ecological shifts is detected rather than averaged away.

### 2.3. Proposed Mode of Action: Decoupling Noise from Signal

The rationale for rigorous fractionation extends beyond hygiene. We hypothesise that crude faecal suspensions act not only as microbial carriers but also as vehicles for soluble bioactive material of donor origin, and that this material can transiently amplify mucosal inflammation at the moment of engraftment. Mechanistic studies of washed preparations provide the most granular available support for this idea [35,36]. Untargeted metabolomic profiling of the washing process has been reported to show selective depletion of specific soluble mediators [22,37], and multi-omic profiling of washed preparations has been reported in Crohn’s disease [35]; the resulting hypothesis is that the preparation behaves as an immunological sieve. We treat the following four mechanisms as hypotheses of decreasing evidential strength rather than as established facts.

#### 2.3.1. Leukotriene B4 (LTB4)

LTB4 is a potent neutrophil chemoattractant whose requirement for neutrophil swarming at sites of cell death has been demonstrated in vivo by intravital imaging in mice, where swarming depends on LTB4 and integrins [38]. The hypothesis for transplantation is a two-step extrapolation: donor-derived LTB4 in a crude suspension would ligate BLT1 on host neutrophils, recruit a neutrophil swarm to the mucosal interface, and generate a locally oxidative environment unfavourable to obligate anaerobes before they can adhere. In a single-centre comparison of washed versus manually prepared transplantation, untargeted liquid chromatography–mass spectrometry (LC-MS) identified LTB4, corticosterone, and prostaglandin G2 among the metabolites removed by repeated washing [22]; the quantification was performed on the preparation itself rather than on the recipient mucosa, no percentage reduction has been reported for LTB4, and we are not aware of independent replication in an unrelated cohort. The finding should therefore be read as a measured difference between two preparation methods in one study, not as a demonstrated effect on neutrophil recruitment in recipients.

#### 2.3.2. Prostaglandin E2 (PGE2)

PGE2 modulates epithelial permeability. In Caco-2 monolayers, PGE2 promotes Ca^2+^-mediated disruption of the epithelial barrier through EP1 and EP4 receptors [39]. On this basis, it has been proposed that excessive donor-derived PGE2 in a crude suspension could transiently loosen tight junctions and permit luminal antigen leakage into the lamina propria. Washed preparation removes a substantial fraction of this lipid together with the rest of the supernatant [22]. As with LTB4, the receptor-level evidence comes from an epithelial cell line rather than from transplanted mucosa, and the contribution of donor-derived PGE2 to post-transplant permeability has not been isolated experimentally.

#### 2.3.3. Glucocorticoid Metabolites: A Rodent-Derived Hypothesis

Faecal matrices contain measurable fractions of unconjugated glucocorticoids and their downstream catabolites [40]. In humans, cortisol is eliminated mainly as hepatic metabolites in urine, with a smaller biliary and faecal fraction, so that stool contains both cortisol and its downstream metabolites [40,41]. The mechanistic evidence for a permeability effect, however, is rodent-derived: in mice, corticosterone mediates stress-related increases in intestinal permeability in a region-specific manner, and luminal glucocorticoid exposure has been reported to modulate mucosal immune homeostasis [42]. Corticosterone is the principal glucocorticoid in rodents, whereas cortisol is the principal bioactive glucocorticoid in humans; the two are not interchangeable, and data obtained with corticosterone in mice cannot be transferred directly to human donor stool. The hypothesis for human transplantation is therefore narrower: that human donor stool contains measurable cortisol and cortisol metabolites, that their concentration may vary between donors, and that they could contribute to transient mucosal immune modulation after transfer. Because the steroid fraction is lipophilic, liquid-phase washing is expected to deplete it together with other supernatant components, but human data on faecal cortisol transfer and its clinical consequences are scarce. We therefore present this mechanism explicitly as a rodent-derived preclinical hypothesis that requires clinical-grade quantification before it can be assigned any weight.

#### 2.3.4. The Virome and the Top-Down Control Hypothesis

Beyond metabolite removal, the fate of the viral fraction during physical fractionation is not settled: free virions are small enough to partition with the discarded liquid phase, and washing has been reported to reduce the viral content of the preparation [22], whereas phages carried as prophages within retained bacterial cells would not be separated by size. Bacteriophages are the most abundant component of the stool viral fraction [43,44]. Phage transfer during FMT has been associated with treatment outcome in rCDI [45], and this association has motivated the hypothesis that phages exert top-down ecological control by targeting specific pathobionts in the recipient gut. The causal step—that phage-mediated predation improves clinical outcome—has not been demonstrated prospectively in humans receiving washed preparations, and the relevant trials have not been designed to separate phage effects from bacterial effects. We therefore classify phage-driven top-down control as a mechanistically plausible but unproven hypothesis.

Taken together, these four mechanisms constitute the components of what we call immunological silence: the hypothesis that a washed preparation presents its bacterial payload to a mucosa that is less acutely provoked, and that this transient reduction in inflammatory signalling is a prerequisite for the seed to overcome colonisation resistance. The two elements of this hypothesis differ in evidential status. Reduction of soluble mediators in the preparation is directly measured [22,37]; reduction of host inflammatory activation and consequent improvement in engraftment in humans are inferred, and no study has yet measured mucosal neutrophil recruitment, macrophage polarisation, or engraftment efficiency as a function of the soluble load of the administered product. This is the single most important experiment in the field, and we return to it in Section 6.

### 2.4. Pharmaceuticalisation: Quantified Dosing

A substantial contribution of WMT to the pharmaceuticalisation of microbiome therapy is the adoption of counted dosing. In conventional FMT, dose is often defined by the wet weight of donor stool (typically 30–50 g), a metric that correlates poorly with microbial load because water and fibre content vary widely between donations [22]. WMT instead anchors dose in bacterial enumeration. Under the methodological consensus established for washed preparation, a therapeutic unit is defined as a specified number of bacteria, and clinical reports express dose in multiples of that unit (for example, 1.0 × 10^13^ bacteria per unit) [22,37]. We report the unit definition as given in that source and direct readers to the original specification, because the definition has been refined as the methodology has matured and a second consensus statement has since been issued [34,46]. Count-based dosing reduces the variability inherent in weight-based dosing, permits reproducible dose–response investigation, and provides a QC benchmark [22,34]. It does not, however, resolve the deeper problem that bacterial counts quantify biomass rather than biological activity, a point developed in Section 2.9.2.

### 2.5. The Global Landscape of Microbiota Processing

**Figure 1:**
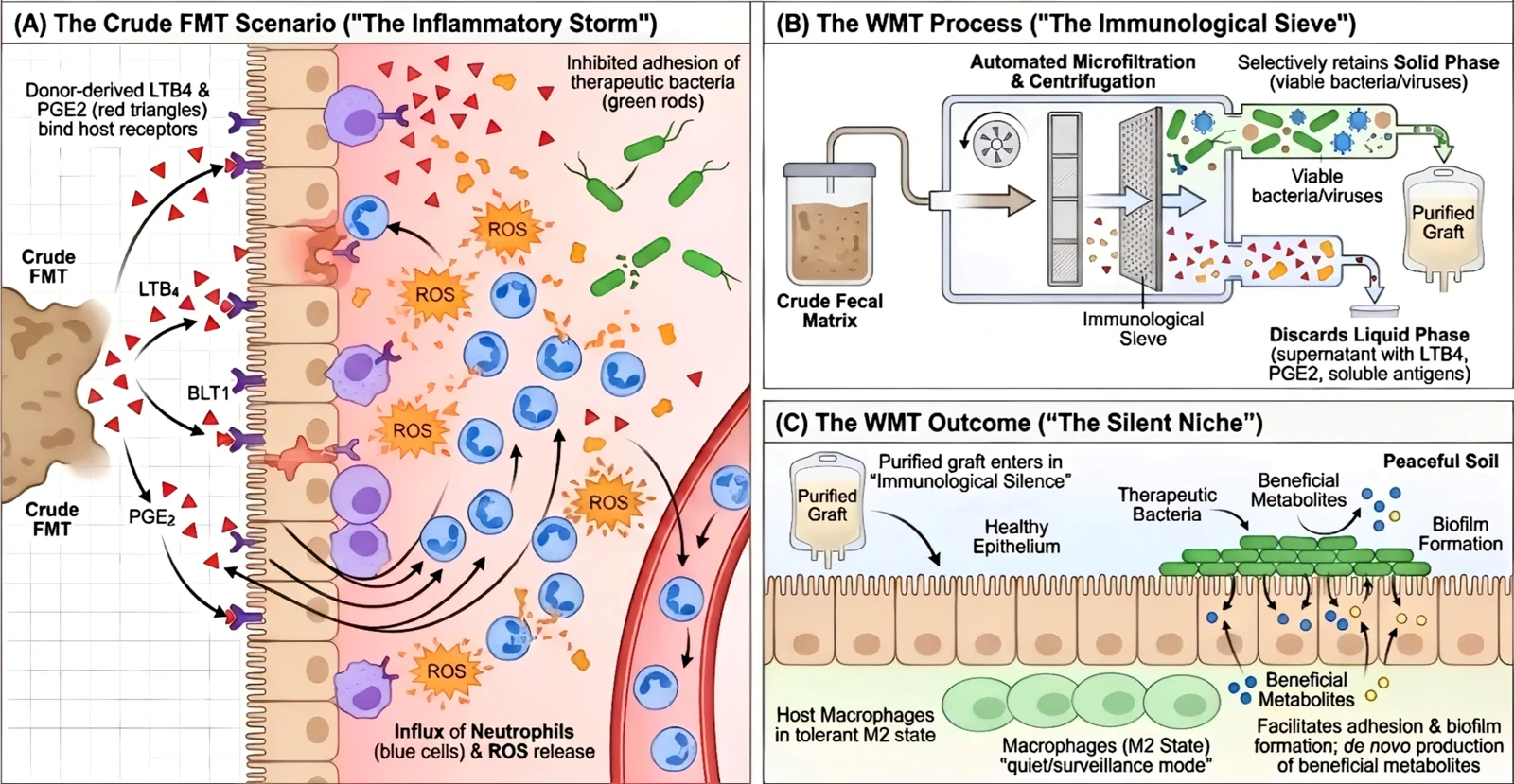
Conceptual illustration of the immunological sieve hypothesis. (**A**) Crude FMT: transfer of an undefined faecal matrix delivers both a viable microbial payload (green rods) and soluble bioactive material, including LTB4 and PGE2 (red triangles), which are hypothesised to ligate host receptors such as BLT1 and to recruit neutrophils (blue cells), generating a locally oxidative environment that may impair colonisation by obligate anaerobes. (**B**) Washed preparation: automated microfiltration combined with repeated low-speed differential centrifugation separates the solid phase (viable bacteria and retained virus-sized particles) from the liquid phase containing soluble mediators, which is discarded. (**C**) Proposed outcome: in the absence of an acute soluble inflammatory stimulus, mucosal macrophages are hypothesised to retain a tolerogenic phenotype, lowering the threshold for colonisation. This figure is a conceptual schematic illustration intended to summarise the hypothesised mechanisms and relationships discussed in the text; it is not derived from primary experimental, quantitative, or imaging data, no quantitative axis or measured curve is implied, and panels (**A**,**C**) depict hypotheses rather than observations.

Globally, microbiota processing methodologies occupy a spectrum that trades purity against diversity:

Minimal processing (for example, early whole-stool protocols) prioritises retention of the complete stool matrix and underlies the great majority of published FMT indications [47], but it also transfers host-derived inflammatory and antigenic material [3,20].
Spore purification (for example, the ethanol-treated product SER-109, marketed as VOWST) uses chemical treatment to isolate spores of the phylum Bacillota (formerly Firmicutes). It achieves high purity and a shelf-stable oral formulation, but sacrifices non-spore-forming commensals, including members of the phyla Bacteroidota and Verrucomicrobiota [48,49].
Quantitative fractionation (for example, WMT) occupies an intermediate position. By using physical rather than chemical separation, it retains a broader bacterial consortium, including Gram-negative taxa, while depleting soluble mediators and also removing virus-sized particles [22]. Whether this configuration confers superior colonisation resilience in inflammatory disease is a hypothesis that has not been tested head to head against spore-based products (Figure 1) [22,50].

### 2.6. Evidence That Complicates the Framework: Two Findings We Meet Head on

Any framework that classifies the soluble fraction as noise must confront two clinical observations that point in the opposite direction. We address them here rather than leaving them implicit, because both are familiar to readers of this field.

Sterile faecal filtrate transfer. Ott and colleagues reported that bacteria-free filtrates of donor stool, prepared by sequential centrifugation and filtration, resolved recurrent CDI in a small series of patients, with resolution of diarrhoea and no recurrence during follow-up [51]. In the taxonomy used here, a filtrate is essentially the fraction we label as noise, and yet it appeared to be therapeutic in that setting. The apparent contradiction is instructive rather than fatal. First, filtrate transfer does not remove everything that fractionation removes: it retains phage-sized particles, bacterial membrane vesicles, soluble proteins, and metabolites, so that what we collectively call the liquid phase is itself a heterogeneous mixture containing potentially therapeutic components. Second, the therapeutic requirement differs between indications. In rCDI, the decisive step may be prompt restoration of bile-acid-mediated colonisation resistance, which can in principle be achieved through soluble effectors and phage predation on *C. difficile* without durable engraftment of a bacterial consortium [52]. Third, the original report was small and uncontrolled, and we are not aware of adequately powered replication, particularly in chronic inflammatory disease. The defensible inference is therefore narrow but important: the soluble fraction is not uniformly deleterious, and the claim that removing it is beneficial must be established function by function, mediator by mediator, and indication by indication. A washed preparation and a sterile filtrate are not interchangeable, and neither is a functional subset of the other.

A spore-based product succeeded in phase 3, while whole-microbiota approaches have been inconsistent. Ethanol-treated spore preparations discard most of the donor ecosystem, including the Gram-negative guilds and much of the viral fraction (Section 2.7), yet the licensed spore product met its primary endpoint in a randomised phase 3 trial in recurrent CDI, reducing 8-week recurrence to 12.4% versus 39.8% with placebo [49,53]. In parallel, whole-microbiota transfer has produced inconsistent results in chronic disease, including UC [9,10,15,50]. This pattern is difficult to reconcile with a simple reading of the subtractive paradigm in which more diversity is better. Our interpretation is that the two settings impose different requirements. Where a single ecological function has been lost and the target is a single organism, a narrow functional implant can be sufficient and may even be preferable, because it is reproducible and shelf-stable and does not transfer donor-derived cellular risk. Where multiple niches and functions are simultaneously disrupted and the host immune compartment is itself a driver of pathology, a narrow implant is less likely to suffice, and the trade-off between taxonomic breadth and procedural risk becomes relevant. On this reading, subtractive preparation is not a universal improvement over chemical selection or over crude transfer; it is a strategy whose value is indication-dependent and, at present, unproven in precisely the indications where it is most attractive. We regard this as the strongest argument for the comparative trial designs proposed in Section 6.

### 2.7. Selection Philosophies: Chemical Inactivation Versus Physical Separation

A fundamental divergence in microbiome engineering lies in the method used to remove unwanted material: chemical inactivation versus physical separation. This is not merely a procedural difference; it determines the ecological architecture of the product. Throughout this review, we use the current validly published phylum names alongside the familiar ones at first mention, following the valid publication of prokaryotic phylum names [54].

The cost of chemical purity. Spore-based products such as SER-109 (VOWST) use ethanol treatment to eliminate vegetative organisms, followed by filtration to remove solids and residual ethanol [48,49]. Ethanol is effective against vegetative bacteria and enveloped viruses, but non-enveloped viruses are substantially more resistant to alcohol [55], and it is not therefore a cross-kingdom sterilisation step; published validation of purified spore manufacture has focused on vegetative bacteria and selected bacterial pathogens rather than on systematic profiling of the viral and fungal metagenome after treatment [48]. We therefore describe the effect as selective depletion or disruption of ethanol-sensitive, non-spore-forming fractions rather than as inactivation of the viral and fungal kingdoms; the precise viability and taxonomic fate of non-bacterial kingdoms in purified spore products remain incompletely profiled and require dedicated metagenomic study. Within the bacterial kingdom, taxa depleted by ethanol treatment include the Gram-negative guilds of the phyla Bacteroidota (formerly Bacteroidetes) and Verrucomicrobiota (formerly Verrucomicrobia), which are important producers of propionate and succinate and contributors to mucus-layer maintenance. Because Actinomycetota (formerly Actinobacteria) are Gram-positive, they cannot be grouped with the Gram-negative guilds; where Actinomycetota are reported as ethanol-sensitive, the mechanism is likely to reflect sporulation status rather than Gram-stain architecture. Accordingly, spore-based products are better described as functional implants that restore specific metabolic capacities, such as bile-acid 7α-dehydroxylation, than as holistic ecosystem replacements (Table 1) [48,49,52].

**Figure 2:**
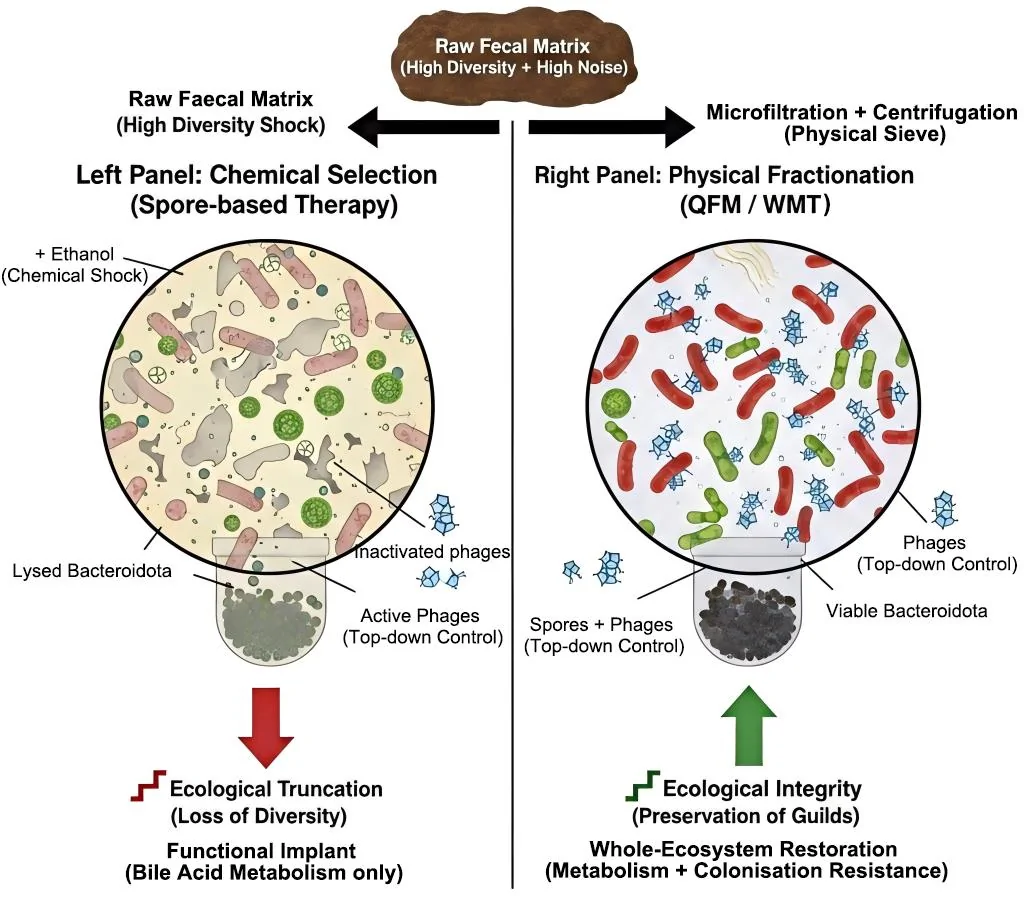
Conceptual comparison of chemical inactivation and physical separation. (**Left**) Chemical inactivation: ethanol treatment acts as a chemical stressor, isolating spore-forming Bacillota (green) while depleting non-spore-forming taxa, including members of the Bacteroidota and Verrucomicrobiota (red, fragmented), and reducing the detectable bacteriophage fraction (blue). Only inactivated or fragmented phages are depicted in this panel. (**Right**) Physical separation: differential centrifugation and microfiltration decouple the solid from the liquid phase, retaining viable Gram-negative guilds and removing soluble mediators together with virus-sized particles. This figure is a conceptual schematic illustration intended to summarise the mechanisms and relationships discussed in the text; it is not derived from primary experimental, quantitative, or imaging data, and the relative sizes of the depicted populations do not represent measured abundances.

The rationale for physical preservation. In contrast, QFM/WMT strategies apply physical sieving, namely differential centrifugation and microfiltration, to decouple the biotic phase from the soluble matrix without chemically compromising cellular integrity. In principle, this preserves Gram-negative commensals, which are among the principal drivers of colonisation resistance through nutrient competition and niche occupation, although the viral fraction is only partially retained, because virus-sized particles are removed together with the soluble phase [22,56]. Whether phages provide top-down control that prevents pathobiont blooms remains a matter of hypothesis, as discussed in Section 2.3.4. Chemical selection offers a simplified, shelf-stable product; physical separation offers a broader, more ecologically complex product whose incremental clinical value is not yet established (Figure 2) [22,45].

**Table 1:** The ecological trade-off between chemical selection and physical fractionation of donor microbiota. Descriptions refer to the manufacturing principle of each modality. The mechanism of action of the licensed spore product has not been established. Mediator removal has been quantified for washed preparation in a single-centre comparison, and the clinical consequences of that removal remain inferred.

Ecological Feature	Chemical Selection (e.g., SER-109/VOWST)	Physical Fractionation (e.g., QFM/WMT)
Selection logic	Chemical stress: ethanol treatment eliminates vegetative organisms and isolates resistant spores, followed by filtration of solids and residual ethanol.	Physical sieving: differential sedimentation combined with multi-stage microfiltration segregates intact cells from the soluble matrix, without a chemical density gradient.
Impact on Gram-negative taxa	Depletion of non-spore-forming Gram-negative guilds, including members of the Bacteroidota and Verrucomicrobiota.	Preservation of vegetative Gram-negative taxa that contribute to nutrient competition and mucus-layer maintenance.
Viral fraction	Substantial reduction of the detectable bacteriophage fraction; validation has focused on bacterial pathogens rather than on viral profiling [48].	Substantial reduction of virus-sized particles together with the soluble phase [22]; whether phages provide top-down control of pathobionts is unproven.
Ecological output	Taxonomically truncated consortium limited to spore-forming Bacillota; shelf-stable oral formulation.	Consortium with greater bacterial taxonomic breadth; donor-dependent and not specifiable a priori.
Proposed therapeutic mechanism	Restoration of defined metabolic modules (for example, bile-acid 7α-dehydroxylation); the licensed product’s mechanism of action has not been established.	Niche occupation and competitive exclusion, together with reduction of soluble mediator load; the link to clinical benefit is inferred.
Evidence for clinical efficacy	Randomised phase 3 evidence in prevention of recurrent CDI (8-week recurrence 12.4% versus 39.8% with placebo) [49,53].	Observational and single-centre evidence in rCDI and UC; no randomised head-to-head comparison with a spore-based product [22,37,50].
Primary proposed indication	Prevention of recurrent CDI after antibacterial treatment; single-target restoration where a defined metabolic pathway suffices.	Complex, multi-factorial pathology (for example, UC, graft-versus-host disease); hypothesis-generating at present.
Regulatory route	Licensed biological product (BLA in the United States); GMP manufacture and lot release required [53,57].	Human tissue or medical procedure under enforcement discretion in several jurisdictions; licensure pathway unresolved [32].

This table is a comparative summary of published information. Quantitative values are reproduced from the cited sources and are not directly comparable across rows or columns.

### 2.8. The Purification Trade-Off: Soluble Mediators Versus Regenerative Capacity

The concession. Any washing step removes material that may be beneficial. The supernatant of donor stool is a reservoir of molecular signals, including short-chain fatty acids (SCFAs), secondary bile acids, antimicrobial peptides, and mucus-associated proteins, that support epithelial barrier function and niche establishment [58,59]. Removing the soluble fraction therefore removes potential trophic factors as well as potential inflammatory triggers. We argue that in acutely inflamed recipients, the short-lived benefit of transferring these metabolites is likely to be outweighed by the acute risk of co-transferring soluble pro-inflammatory mediators [22,59], but this trade-off has not been quantified in a controlled comparison and may differ between indications.

**Figure 3:**
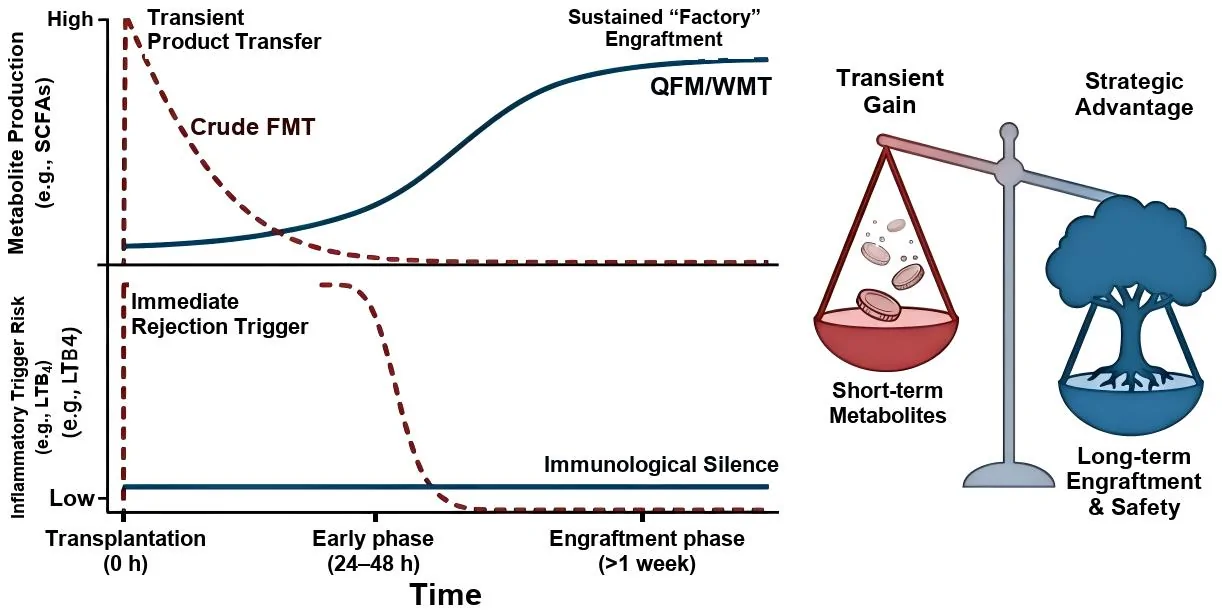
Conceptual framework of the purification trade-off. The curves in this figure are illustrative and conceptual; no data underlie them, and no quantitative value on either axis, nor any specific time point, should be inferred. The upper panel depicts hypothesised functional output (for example, in situ metabolite production) and the lower panel the hypothesised risk of acute inflammatory triggering, as a function of time after transplantation. A crude preparation (dashed line) is hypothesised to deliver an immediate metabolite bolus together with a concurrent inflammatory stimulus, with an early decline in function if the graft is not retained. A washed preparation (solid line) is hypothesised to start from a lower functional baseline because soluble products have been removed, but to rise towards a sustained plateau if engraftment succeeds. The plateaus, crossover points, and time constants shown are schematic. This figure is a conceptual schematic illustration intended to summarise the mechanisms and relationships discussed in the text; it is not derived from primary experimental, quantitative, or imaging data.

The counter-argument: factory versus product. The soluble metabolites delivered by a crude transplant are transient, with absorption half-lives measured in minutes to a few hours in the human colon [59], whereas a successfully engrafted microbiota represents a regenerative capacity capable of sustained production. On this reasoning, the primary goal of transplantation is not the acute delivery of metabolites but the durable engraftment of their producers [60]. The transient loss of exogenous SCFAs would then be an acceptable cost for long-term functional restoration (Figure 3). We note that this argument presupposes that the removed metabolites are not themselves required for early engraftment, which remains untested.

The proposed mechanism, restated. The counter-argument depends on the claim that the crude matrix carries disproportionate risk. We hypothesise that, alongside beneficial signals, the crude matrix contains higher concentrations of soluble mediators, including LTB4, PGE2, and cortisol metabolites, as well as unbound cytokines, and that these molecules can transiently amplify host immune surveillance and thereby reduce the probability that a fragile graft establishes a foothold [22]. If correct, washing would create a window of reduced inflammatory signalling in which bacteria can adhere and proliferate without an immediate neutrophil-dominant response [38], after which the physiological concentrations of beneficial metabolites would be restored in situ [56]. This chain of reasoning contains one directly measured step (lower mediator concentrations in the preparation), one indirectly supported step (mediator concentrations high enough to matter biologically), and one untested step (improved engraftment as a consequence). We return to this decomposition in Section 5.1.

### 2.9. Translational Governance: Regulation, Potency, Release Specifications, and Surveillance

The clinical adoption of purified microbiota demands parity with modern biopharmaceutical standards. Washed preparation mitigates acute infusion-related reactivity by physically discarding soluble pro-inflammatory mediators, but it does not bypass the fundamental CMC vulnerabilities shared by all donor-derived biological grafts: batch heterogeneity, donor phenotype drift, and the risk of transferring occult pathogens or horizontally transferable resistomes [32,61]. The following four subsections set out the frameworks that would be required for QFM/WMT to operate as a regulated product rather than as an unstandardised procedure.

#### 2.9.1. Regulatory Taxonomy and GMP Manufacturing

The global regulatory status of faecal microbiota preparations is bifurcated. Agencies such as the FDA classify microbiome-based therapeutic entities under the Center for Biologics Evaluation and Research as biological products, requiring full investigational new drug (IND) and biologics licence application (BLA) compliance, and the licensure of RBX2660 (REBYOTA in November 2022) and SER-109 (VOWST in April 2023) demonstrates that this route is workable for defined products [32,53,57]. In contrast, unlicensed FMT has generally been used in the United States under a policy of enforcement discretion for rCDI unresponsive to standard therapy, a pathway that does not require product licensure [32]. In other jurisdictions, donor-derived preparations have been handled through hospital exemptions, tissue-and-cell directives, or medical-technology frameworks [32]. The practical implication is that the regulatory asymmetry, rather than the underlying biology, is currently a major determinant of which modality reaches the clinic first.

For WMT to sustain biopharmaceutical legitimacy, manufacturing would need to move fully into current Good Manufacturing Practice (cGMP) environments, for example Grade C processing within Grade A biosafety enclosures or fully closed automated single-use fluidics. Such an architecture requires validated cleaning procedures, demonstrated absence of cross-batch contamination, continuous environmental particulate monitoring, and validated cold-chain cryopreservation under documented digital audit trails [32,33].

#### 2.9.2. A Multidimensional Potency Matrix Beyond Microbial Count

Establishing a validated potency assay is a major hurdle for donor-derived consortia. Release testing for licensed biologics must incorporate quantitative biological assays that reflect the intended mechanism of action. Because washed preparation is proposed to act through several mechanisms—pathogen exclusion, bile-acid transformation, barrier support, and immune conditioning—reliance on total particle count or on generic membrane-integrity dyes alone cannot satisfy a formal potency requirement [33,62]. A regulatory-grade potency matrix would combine structural viability with functional read-outs, including the following.

Targeted enzymatic conversion kinetics. Ex vivo assays quantifying the catalytic rate of the metabolic biotransformations considered responsible for efficacy. For rCDI and metabolic indications, release lots could be assayed by targeted liquid chromatography–tandem mass spectrometry (LC-MS/MS) for the conversion of conjugated primary bile acids (for example, taurocholic acid) to secondary bile acids (for example, deoxycholic acid) through bile salt hydrolase and 7α-dehydroxylase activity within a standardised reference interval [52,63].

Cell-based immunomodulatory bioassays. Standardised co-culture reporter assays using human intestinal epithelial cell lines (for example, Caco-2 or HT29-MTX) or macrophage and dendritic-cell platforms, measuring dose-dependent suppression of lipopolysaccharide-induced tumour necrosis factor (TNF) or interleukin-8 secretion, or modulation of nuclear factor-κB (NF-κB) and STAT3 signalling [33].

Microcalorimetric metabolic vigour. Real-time isothermal microcalorimetry measuring total thermodynamic metabolic flux and heat-generation kinetics after reconstitution in standardised media, providing an unbiased index of overall bacterial metabolic activity before cryopreservation and after thawing [33].

None of these approaches has yet been qualified as a release assay for a donor-derived product, and the field lacks consensus reference materials, which is the principal obstacle to cross-centre comparability. Priority 2 of the research roadmap (Section 6) addresses this directly.

#### 2.9.3. Lot-to-Lot Release Specifications and QC Thresholds

To maintain product uniformity, every manufactured batch would need to pass predefined, validated release criteria before delivery to a patient. A defensible specification set includes the following parameters, which we present as a proposed framework rather than as an established standard.

Viable biomass and viability threshold. Absolute cell concentration quantified by calibrated flow cytometry (for example, a lower limit of 1.0 × 10^13^ total cells per unit) with post-thaw membrane integrity above a pre-specified threshold (for example, at least 80% viable, metabolically active cells) [34].

Depletion limits for soluble host contaminants. Quantitative limits for residual host-derived pro-inflammatory small molecules measured by validated immunoassay or mass spectrometry. Illustrative target specifications include residual LTB4 below 50 pg/mL, residual PGE2 below 75 pg/mL, and greater than 90–95% clearance of these analytes and of free bioactive glucocorticoids relative to the crude donor input. Total protein and free haemoglobin should also be kept below pre-specified limits once these have been established, and the values proposed here are ours rather than consensus values [22,37]. We stress that these numerical limits are illustrative proposals for discussion, not consensus values.

Pathogen and adventitious agent screening. Mandatory negative panels by high-sensitivity quantitative PCR and culture for multidrug-resistant organisms (MDROs), including carbapenem-resistant Enterobacterales, vancomycin-resistant enterococci, extended-spectrum β-lactamase (ESBL)-producing species, and methicillin-resistant *Staphylococcus aureus*; testing for enteric protozoa, microsporidia, and helminths; and viral screening including severe acute respiratory syndrome coronavirus 2, norovirus, rotavirus, adenovirus, cytomegalovirus, and Epstein–Barr virus [19,27,64,65].

Metagenomic purity and compositional stability. Shallow or deep shotgun metagenomic sequencing of manufacturing retain aliquots to benchmark taxonomic boundaries, to confirm the absence of pathobiont blooms (for example, sudden expansion of Enterobacterales or *Fusobacterium*), and to demonstrate stability of core obligate anaerobic guilds (Bacteroidota, Bacillota) within pre-established confidence intervals across donations from the same qualification cohort [33,48].

Donor-derived products remain biologically tethered to donor variability even when the manufacturing process is highly standardised. Unlike a clonal LBP, a donor-derived product has a composition that cannot be specified a priori, and batch-to-batch consistency must therefore be demonstrated statistically rather than by identity testing.

#### 2.9.4. Serious Adverse Events, Multidrug Resistance, and Long-Term Vigilance

Washed preparation must not be presented as a substitute for donor screening. In 2019, two immunocompromised recipients of FMT from a single donor developed invasive ESBL-producing *Escherichia coli* infection, with one death, and whole-genome analysis was consistent with donor-derived transmission [64]. The FDA issued a safety alert in June 2019 [65] and in March 2020 reported additional severe and life-threatening infections caused by enteropathogenic *E. coli* and Shiga-toxin-producing *E. coli* linked to a stool bank, which led to mandatory nucleic acid amplification testing of donor stool for these organisms together with quarantine of untested product [66,67]. These events have a direct implication for the present framework: centrifugal washing sediments intact bacterial biomass and therefore cannot selectively remove intact MDROs if they are present in the donor material. Screening failures are upstream of the washing step, and no physical fractionation step can be assumed to compensate for them.

What washing can plausibly achieve is a reduction in acute, non-infectious procedure-associated reactivity, such as post-infusion fever and abdominal discomfort, through clearance of cell-free pyrogens, unbound endotoxin, and inflammatory eicosanoids from the discarded supernatant. That benefit is mechanistically reasonable and partly supported by observational comparisons of washed and manual preparation [22,37]; it is not evidence that washing reduces the risk of transmitting cellular pathogens.

Two further liabilities require explicit governance. First, unlike clonal LBPs, undefined donor consortia harbour variable mobilomes, including integrative conjugative elements, plasmids, and prophages carrying antimicrobial resistance genes or cryptic virulence factors; even clinically healthy donors may carry subclinical resistome signatures that could be transferred to a dysbiotic recipient niche [33,68]. Second, multi-kingdom donor transfer carries the hypothetical risk of transmitting phenotypes with long latency, including subclinical oncogenic determinants such as *pks*-positive *E. coli* producing colibactin [69] or enterotoxigenic *Bacteroides fragilis* [70]. Addressing these liabilities would require institutionalised post-market pharmacovigilance, comprising mandatory longitudinal registries recording recipient outcomes at standardised checkpoints (for example, 6 months, 1, 5, and 10 years) for new-onset chronic illness, autoimmune disease, metabolic change, and malignancy; long-term cryogenic archiving of donor-product retains paired with recipient pre- and post-transplantation samples, to permit trace-back metagenomic and whole-genome sequencing should an adverse outcome emerge; and resistome mapping as a standard gatekeeper within donor qualification, excluding material with elevated baseline resistome indices or mobile transposon-associated resistance determinants [33,62,68].

## 3. The Additive Paradigm: Ecosystem Reconstruction with Defined Consortia

### 3.1. Principles of Assembly: From Stochastic Transfer to Ecological Engineering

Whereas QFM/WMT represents optimisation of a top-down approach by purifying a natural ecosystem, the additive paradigm pursues bottom-up reconstruction through defined LBPs [23,71]. This transition moves beyond transfer of an undefined ecosystem to administration of a characterised pharmaceutical [62]. LBP development is, in effect, applied community ecology. The limitations of single-strain probiotics have established that many functions are emergent properties of networks rather than of individual organisms [33,72], and contemporary design therefore uses ecological network analysis to identify keystone taxa, that is, species that function as architectural linchpins irrespective of their absolute abundance [72]. The objective is to assemble a consortium that occupies defined nutrient and spatial niches and thereby excludes pathogens through competitive exclusion and resource sequestration [52,62].

### 3.2. Mechanistic Distinctness: Ecological Filtering Versus De Novo Synthesis

Two platforms illustrate the range of engineering philosophies available.

Ecological filtering (the SER-109/VOWST model). SER-109, licensed in the United States on 26 April 2023 and marketed as VOWST (fecal microbiota spores, live-brpk), is a donor-derived product in which ethanol treatment eliminates vegetative organisms and isolates a consortium of purified Bacillota spores; each capsule contains 1 × 10^6^ to 3 × 10^7^ spore colony-forming units, and the licensed dose is four capsules daily for three consecutive days [49,53]. In the pivotal phase 3 trial, recurrence of CDI at 8 weeks was 12.4% with SER-109 versus 39.8% with placebo [49]. The licensed indication is prevention of recurrence of CDI in adults following antibacterial treatment for recurrent CDI; the product is not indicated for treatment of active CDI, and the mechanism of action of the licensed product has not been established [53]. Restoration of the secondary bile-acid pool is a well-supported mechanistic axis in preclinical and correlative work, since re-establishing the enzymatic machinery that converts primary to secondary bile acids creates a microenvironment inhibitory to *C. difficile* spore germination and vegetative outgrowth [49,52,63]; it should not, however, be presented as settled. The in vivo response is increasingly regarded as multifactorial, plausibly involving competitive nutrient exclusion, niche competition, and mucosal cross-talk. This platform nonetheless demonstrates that therapeutic restoration may not require a full microbiome reset, but rather the targeted reinstatement of defined functional modules.

De novo synthesis (the VE303 model). VE303 is a rationally defined consortium of eight clonal Clostridia strains manufactured from master cell banks under GMP conditions, which bypasses donor material entirely [73]. Its clinical strategy illustrates an ecological principle: engraftment requires opportunity. In the phase 2 trial, vancomycin pretreatment was used to perturb the indigenous microbiota and create a transient open niche before consortium administration, and the consortium expanded and persisted in a dose-dependent manner [63,73]. That study provided significant efficacy signals in prevention of recurrent CDI, but definitive validation is pending: the phase 3 RESTORATiVE303 trial passed a protocol-specified interim analysis for futility and was permitted to continue without modification, and it has not yet reported its final primary endpoint [74,75]. The VE303 programme therefore provides a conceptual proof of principle for synthetic consortium therapy, while the extent to which antibiotic preconditioning can reliably overcome colonisation resistance remains to be confirmed by completed phase 3 data.

### 3.3. The Precision Promise and the Engraftment Barrier

Defined consortia offer advantages that donor-derived therapies cannot match. Unlike washed preparation, which retains uncharacterised biotic interactions and viral elements, a synthetic LBP provides compositional definition, which facilitates genomic exclusion of antibiotic resistance genes, lot-to-lot consistency, and the deliberate engineering of function, such as induction of regulatory T cells in inflammatory bowel disease [76] or potentiation of checkpoint inhibitor responses in oncology [62,77]. The corresponding limitation is that definition is achieved by simplification. Communities assembled from a small number of strains lack the cross-feeding networks of a native ecosystem and are correspondingly vulnerable to competitive exclusion by the resident microbiota [78].

### 3.4. Divergent Trajectories and Convergent Futures

The complexity–control trade-off. The divergence between WMT and LBPs is best understood as a trade-off between holistic ecological restoration and precision functional modulation [62]. Washed preparation retains a broader bacterial consortium than ethanol-treated spore preparations, including Gram-negative guilds [22,48], and this diversity may provide functional redundancy that allows simultaneous occupation of multiple disrupted niches [79]. Systematic comparisons of whole-microbiota strategies against defined products in UC do not currently exist: no randomised head-to-head trial has compared a washed preparation with an LBP, and published remission rates for whole-microbiota transfer in UC derive from heterogeneous trials that differ in donor selection, preparation, delivery route, endpoint definition, and era [9,10,15,50]. Any statement that whole-microbiota strategies achieve superior remission rates should therefore be presented as an indirect and confounded comparison rather than as an established finding.

Quantitative safety data and their limits. Regarding safety, national stool-bank series report fewer short-term transplantation-related adverse events with the automated washed process than with manual preparation (for example, 7.2% versus 35.5% in ulcerative colitis and 4.0% versus 21.7% in Crohn’s disease, with post-transplant fever falling from 19.4% to 2.7%), and the events described were predominantly mild and self-limiting [22,37]; in a nationwide 24-hospital real-world cohort of 216 washed-transplantation courses, the overall adverse-event rate was 8.3% [80]. In the pivotal phase 3 trial of SER-109, solicited adverse reactions reported in at least 5% of recipients were abdominal distension (31.1%), fatigue (22.2%), constipation (14.4%), and chills (11.1%), predominantly mild to moderate, and no serious adverse events were considered product-related among 349 recipients [49,53]. A systematic review of FMT-related adverse events reported adverse events in approximately 19% of FMT procedures and serious adverse events in approximately 1.4% of patients [81], whereas an earlier systematic review with different ascertainment reported adverse events in 28.5% and serious adverse events in 9.2% of recipients [82]. These figures derive from different designs, populations, follow-up durations, and ascertainment methods, and they are not directly comparable; direct comparative quantitative safety data between washed preparation and LBPs are lacking. We present them to make the absence of a clean comparison explicit rather than to imply one.

Three converging directions. First, computational design: bottom-up computational models of microbial interaction networks may eventually allow prediction of a minimal consortium sufficient for a given function [83]. We note that the size of such a consortium cannot be specified in advance; the claim that a consortium of 50 or more strains is “minimal” is internally inconsistent, because minimality is defined by the smallest set that achieves a stated function, and current evidence supports neither a lower nor an upper bound. Second, staged hybrid therapy: using WMT as an ecological reset followed by a defined LBP to install specific functions [62]. Third, postbiotic therapeutics: if the relevant mechanism is purely metabolic, defined metabolites or effector proteins identified through WMT and LBP research could be administered directly, bypassing live organisms altogether [84] (Table 2).

## 4. Ecological Engineering: The Soil-and-Seed Framework

We propose that the dichotomy between subtractive and additive paradigms is a false one, and that the more productive question is how they can be combined. The organising metaphor is agricultural: soil and seed. The framework has three components, to which we add a fourth—substrate—to acknowledge that engraftment depends on host-derived nutrients and mucosal conditions as much as on the organisms themselves.

### 4.1. Phase I: Preparing the Soil (Subtractive Priming)

A field must be cleared before delicate seed is planted. In this framework, washed preparation acts through three mechanisms, each with a different evidential status:

Physical clearance of dysbiotic biofilm and pathogen reservoirs. Mechanistically plausible from the fluid mechanics of lavage and sieving; not directly demonstrated in vivo.
Reduction of soluble mediator load. Directly measured in the preparation [22,37]; the corresponding reduction in mucosal inflammatory signalling in recipients remains inferred.
Lowering of the colonisation-resistance threshold, proposed to create a receptive niche [56]. This is the central and least tested claim of the framework.

We stress that priming is not the same as eradication. Washed preparation does not sterilise the gut, does not remove intact MDROs (Section 2.9.4), and cannot be assumed to open niches that the resident community continues to occupy.

### 4.2. Phase II: Planting the Seed (Additive Engraftment)

Into the primed niche, a defined LBP is introduced. Two ecological principles are invoked:

Priority effects. Early arrivers can gain a durable advantage in community assembly [85]. The magnitude of this advantage in a human adult gut, where the resident community is already structured, is uncertain and likely to be smaller than in the neonatal or post-antibiotic settings in which priority effects have been best documented.
Functional implantation. The LBP supplies a defined activity, such as tumour-antigen cross-reactive immunity [86] or targeted interleukin-10 (IL-10) secretion [87,88], that a generic donor preparation may not reliably provide.

### 4.3. Phase III: The Substrate Problem (Host and Dietary Determinants)

Engraftment is not solely a property of the inoculum. Colonisation resistance is maintained by host physiology as well as by the resident microbiota: microbiota-activated PPAR-γ signalling, for example, restricts the expansion of dysbiotic Enterobacterales by maintaining luminal anaerobiosis [56], and prior pathogen exposure can train the host for microbiota-enhanced resistance [89]. Durable coexistence of donor and recipient strains after FMT likewise depends on the recipient’s ecological context [60]. Dietary substrate availability, mucosal oxygen tension, bile-acid pool composition, and immunoglobulin A repertoire are all plausible determinants of whether an introduced strain persists. Any clinical protocol that omits substrate management is therefore likely to underestimate the requirements for durable engraftment. We include this phase to make explicit that the soil is not only a microbial community; it is also a host environment.

### 4.4. A Thought Experiment, Not a Case Report

The following scenario is explicitly hypothetical. It is constructed to make the framework’s assumptions testable, not to describe an observed clinical course, and no such treatment has been validated in any indication. Consider a patient with moderately to severely active UC refractory to conventional therapy. In the hypothetical phase 1, the patient receives a washed preparation, which removes the soluble fraction, reduces soluble mediator exposure, and is hypothesised to create a more permissive mucosal state [22]. In the hypothetical phase 2, at an interval that would itself require optimisation, an engineered LBP designed to produce butyrate and to secrete an anti-TNF nanobody is administered [88]. The presumed sequence—priming, then engraftment of a defined function, then sustained remission—is a conjecture. No preclinical or clinical study supports a specific interval, and no engineered anti-TNF-secreting consortium has been tested in UC. The only available data concern engineered *Lactococcus lactis* strains in murine colitis models [87,88] and the use of non-engineered consortia in rCDI [49,73].

### 4.5. What Would It Take to Test the Soil-and-Seed Hypothesis?

The framework is falsifiable, and the following design addresses its principal weaknesses. Endpoint definitions should be pre-specified and separated into ecological engraftment, functional engraftment, and clinical outcome.

- Sequencing interval. The interval between priming and seeding should be treated as an experimental variable with at least two levels, justified by the kinetics of mucosal inflammatory resolution after priming rather than by convention. Serial measurement of faecal calprotectin, mucosal cytokine profiles, and recipient soluble mediator concentrations should define the biological window empirically.
- Engraftment read-out. Strain-level engraftment should be quantified by metagenomic strain tracking with unique sequence signatures in the administered consortium [60,63], complemented by metatranscriptomic confirmation of expression of the engineered function and by quantitative measurement of the intended metabolite in faeces and serum. Colonisation resistance should be reported as a composite of residual resident diversity, pathobiont abundance, and ex vivo growth inhibition of an indicator pathogen.
- Control conditions. At a minimum, priming alone, seeding alone, and priming plus seeding must be compared, with blinding of outcome assessment. Without the priming-alone arm, any incremental effect of the LBP cannot be attributed.
- Safety. Combining deliberate ecological disruption with a genetically modified organism introduces risks that neither component carries alone, including chromosomal or plasmid transfer of the engineered construct to resident organisms, unintended persistence of the engineered strain after withdrawal, loss of colonisation resistance against MDROs during the priming window, and the immunological consequences of exposing a disrupted mucosa to a high-dose defined consortium. Containment strategies such as auxotrophy, kill switches, and non-transferable genetic architecture should be mandatory design features rather than optional refinements [90], and protocols should include surveillance for at least five years.
- Regulatory pathway. Because priming and seeding are separate interventions with different regulatory classifications—tissue or procedure versus biological drug—the combination requires either a formal combination-product pathway or a trial design in which the two components are separately authorised. This is a solvable but currently unresolved obstacle to clinical translation [32,33,61].

## 5. Evidence Grading: Supported Findings, Borrowed Mechanisms, and Speculation

Because the framework combines observations of differing quality, we state explicitly which links in the chain have direct human support, which are extrapolated from unrelated model systems, and which are speculative. Table 3 summarises this classification. We regard this decomposition as a substantive part of the contribution: a framework that does not distinguish measured effects from borrowed mechanisms invites over-interpretation, and readers are entitled to see where the boundaries lie.

### 5.1. Direct Human Evidence

Three elements are supported by direct human measurement. First, washed preparation reduces the concentration of soluble mediators, including LTB4, relative to manual preparation, measured in the preparation itself [22,37]. Second, washed preparation is associated with fewer acute procedure-related adverse events than manual preparation in observational comparison [22,37]. Third, defined LBPs can reduce CDI recurrence in randomised trials, with the caveat that the mechanism of action of the licensed spore product has not been established [49,53,73].

### 5.2. Evidence Borrowed from Other Systems

Several mechanistic steps are extrapolated. LTB4-dependent neutrophil swarming has been demonstrated by intravital imaging in mice, not in transplanted human mucosa [38]. PGE2-mediated barrier disruption has been demonstrated in Caco-2 monolayers [39]. Glucocorticoid-mediated permeability changes have been demonstrated with corticosterone in rodents, not with cortisol in humans [42]. Priority effects have been characterised mainly in early-life assembly [85]. Secretion of engineered IL-10 and anti-TNF has been demonstrated in murine colitis [87,88]. Each of these findings is mechanistically informative, and each requires confirmation in the human transplant setting before it can be used to justify a clinical strategy.

### 5.3. Speculation

The following claims are hypotheses without direct supporting data in humans: immunological silence as an operative state after washed transplantation; tolerogenic or M2-polarised macrophage skewing in recipients of washed preparations; phage-driven top-down control of pathobionts in recipients; improved engraftment of a subsequently administered LBP as a consequence of priming; and the clinical superiority of the soil-and-seed sequence over either component alone. We retain them because they generate testable predictions, not because they are established.

### 5.4. Independence and Replication

A further limitation of the current evidence base is concentration. Much of the quantitative work on washed preparation, including the mediator measurements, the dose standard, and the comparison with manual preparation, originates from a single research group and, in several instances, from a single centre [22,34,35,37]. Independent replication of the central quantitative claims—particularly the magnitude of mediator reduction and the relationship between mediator load and clinical or immunological outcomes—is therefore a priority. For a review proposing a general paradigm, we regard the present evidence base as sufficient to justify a hypothesis and a research agenda, but not sufficient to justify a treatment recommendation. We have calibrated the claims in this paper accordingly. Where independent clinical data do exist, for example on long-term safety after FMT, they are cited for the specific questions they address [68].

## 6. Outstanding Questions and a Prioritised Research Roadmap

The framework raises questions that differ greatly in tractability. We order them by the priority we would assign, and we pair each with the experiment or analysis required to answer it. Priority 1 is, in our view, the experiment on which the framework stands or falls.

Priority 1—Dose–response of noise. Does the soluble load of an administered preparation determine mucosal inflammatory activation and engraftment efficiency in humans? This requires a randomised comparison of preparations differing systematically in soluble mediator content, with pre-specified mucosal and ecological endpoints [22,37].
Priority 2—Definition and qualification of a potency assay. Can any functional assay predict clinical or ecological performance across lots? The immediate requirement is a multi-centre ring trial using shared reference materials, without which no release specification can be compared between manufacturers [33,62].
Priority 3—Host determinants of engraftment. Which recipient factors, including diet, bile-acid pool, mucosal oxygen tension, and immunoglobulin A repertoire, predict durable engraftment of an introduced consortium? This is addressable by longitudinal multi-omic sampling in existing LBP and WMT cohorts rather than by new interventional studies [56,60,78].
Priority 4—The non-bacterial kingdoms. Do bacteriophages, fungal taxa, and archaea contribute causally to outcome, or are they passengers? The required design compares preparations matched for bacterial content but differing in viral content, which is now technically feasible through virome depletion and repletion [21,44,45].
Priority 5—The super-donor enigma. Is donor-dependent efficacy attributable to specific keystone strains, to non-bacterial components such as phages or immunoglobulin A, or to host–donor ecological matching? This question is best addressed by re-analysing existing donor–recipient cohorts at strain and virome resolution [16,18,91].
Priority 6—Regulatory convergence. How should a two-step intervention comprising a tissue-classified priming step and a drug-classified seeding step be evaluated? A workable pathway, whether as a combination product or as two separately authorised components, is a prerequisite for any hybrid trial [32,33,61].

## 7. Conclusions

The transition from crude FMT to fractionated preparations and defined consortia marks a methodological maturation rather than a completed therapeutic revolution [92]. Fractionation has standardised preparation, reduced the acute reactivity associated with soluble inflammatory mediators, and introduced counted dosing, thereby creating a bridge to contemporary pharmaceutical practice [22,34]. Defined consortia have, for the first time, delivered licensed, reproducible biological drugs with a characterisable manufacturing route and a defined indication [49,53,57]. Neither approach is sufficient on its own. Fractionated donor material remains biologically variable and cannot be specified a priori, and its mechanism of benefit is inferred rather than established; defined consortia are precise but ecologically fragile and often require antibiotic preconditioning to engraft [73,78].

The framework we propose holds that the two paradigms are complements. Subtractive priming would lower colonisation resistance transiently, and additive seeding would install defined functions into the resulting window. We have deliberately presented this as a hypothesis with an explicit validation roadmap, because the critical experiment—relating the soluble load of a preparation to mucosal immune activation and engraftment in humans—has not been performed, and because the quantitative definitions required to operationalise a signal-to-noise distinction do not yet exist. What is required is not a new metaphor but a set of measurable endpoints: qualified potency assays, agreed release criteria, long-term surveillance registries, and trials designed to separate priming from seeding. If those are delivered, the field will have moved from descriptive microbiology towards a testable engineering discipline. Whether the soil-and-seed sequence improves outcomes is an empirical question, and it should be answered as one.

## Figures and Tables

**Table 2:** The methodological spectrum: subtractive (WMT) versus additive (LBPs). Quantitative safety figures are drawn from the sources indicated and are not directly comparable, because they derive from different designs, populations, follow-up durations, and ascertainment methods. AE: adverse event; MDRO: multidrug-resistant organism.

Feature	Washed Microbiota Transplantation (WMT)	Synthetic Consortia (LBPs)
Therapeutic philosophy	Ecological reset: broad restoration of ecosystem architecture (top-down).	Functional implant: delivery of defined metabolic or immune modules (bottom-up).
Compositional complexity	Broad bacterial diversity; soluble noise depleted and virus-sized particles removed with it, but composition not specified a priori [22,37].	Defined precision: typically ten strains or fewer, restricted to bacteria; deliberately excludes uncharacterised elements.
Ecological robustness	Broader functional redundancy is hypothesised to aid colonisation in complex dysbiosis; unproven in controlled comparison.	Variable. Simplified communities are susceptible to competitive exclusion and may require antibiotic preconditioning to open niches.
Safety profile and its basis	Improved relative to manual preparation on the basis of automated washing that removes cell-free pyrogens, unbound endotoxin, and inflammatory eicosanoids; observational comparisons report fewer acute procedure-related events (7.2% versus 35.5% in ulcerative colitis and 4.0% versus 21.7% in Crohn’s disease), with an overall adverse-event rate of 8.3% in a nationwide 24-hospital real-world cohort [22,37,80]. Washing does not remove intact MDROs and does not replace donor screening.	Based on GMP manufacture, absence of donor material, and full genomic characterisation. In the pivotal SER-109 trial, solicited adverse reactions in at least 5% of recipients were abdominal distension (31.1%), fatigue (22.2%), constipation (14.4%), and chills (11.1%), predominantly mild to moderate; no serious adverse events were considered product-related among 349 recipients. Across FMT studies, adverse events have been reported in approximately 19% of procedures and serious adverse events in approximately 1.4% of patients [81].
Scalability	Moderate to high. Centralised processing, closed-system automation, and lyophilisation allow a single screened donation to supply multiple doses; output is limited by donor eligibility and by lot-to-lot biological variability.	High and reproducible. Clonal master cell banks allow essentially unlimited, fully specified manufacture with defined shelf life.
Regulatory framework	Human tissue or medical procedure in several jurisdictions, often under enforcement discretion; classification as a biological drug remains contested.	Biological drug: requires an investigational new drug application and licensure, with GMP manufacture and validated release testing.
Principal open question	Does reduced soluble mediator load translate into measurably improved engraftment and clinical outcome in humans?	Can defined consortia persist without antibiotic preconditioning, and how large must a consortium be to be self-sustaining?

This table is a comparative summary of published information. Quantitative values are reproduced from the cited sources and are not directly comparable across rows or columns.

**Table 3:** Evidence grading for the mechanistic claims made in this review. “Borrowed from other systems” indicates that the mechanism has been demonstrated in a model system (murine tissue, immortalised epithelial monolayers, or early-life human cohorts) rather than in the human transplant setting. CDI: *Clostridioides difficile* infection.

Claim	Direct Human Evidence	Borrowed from Other Systems	Speculation
Washing reduces LTB4 and other soluble mediators in the preparation [22,37]	Yes (single-centre, preparation-level)	—	—
Washing reduces acute procedure-related adverse events [22,37]	Observational	—	—
Defined LBPs reduce recurrence of CDI [49,53,73]	Yes, randomised	—	Mechanism of the licensed spore product is not established
LTB4 drives neutrophil swarming in transplanted mucosa [38]	—	Mouse intravital imaging	Human transplant setting
PGE2 increases barrier permeability after transplantation [39]	—	Caco-2 monolayers	Donor-derived PGE2 as a clinical driver
Glucocorticoid metabolites modulate recipient immunity [42]	—	Rodent corticosterone	Human faecal cortisol transfer
Phages provide top-down control of pathobionts in recipients [45]	Phage transfer associates with outcome	—	Causal benefit; requires virome-matched designs
Washing produces immunological silence and tolerogenic macrophage skewing	—	—	Entirely hypothesised
Priming improves subsequent LBP engraftment	—	—	Entirely hypothesised; the framework’s central claim
Soil-and-seed sequencing improves clinical outcome	—	—	Entirely hypothesised

This table is a comparative summary of published information. Quantitative values are reproduced from the cited sources and are not directly comparable across rows or columns.

## Data Availability

No new data were created or analysed in this study. Data sharing is not applicable to this article.

## Abbreviations

The following abbreviations are used in this manuscript:

AE	Adverse event
AMR	Antimicrobial resistance
BLA	Biologics licence application
BLT1	Leukotriene B4 receptor 1
BSH	Bile salt hydrolase
CDI	*Clostridioides difficile* infection
CFU	Colony-forming unit
CMC	Chemistry, manufacturing and controls
cGMP	Current Good Manufacturing Practice
CR	Colonisation resistance
DAMPs	Damage-associated molecular patterns
EP	E-prostanoid receptor
ESBL	Extended-spectrum β-lactamase
FDA	United States Food and Drug Administration
FMT	Faecal microbiota transplantation
GMP	Good Manufacturing Practice
GVHD	Graft-versus-host disease
IBD	Inflammatory bowel disease
IL-8	Interleukin-8
IL-10	Interleukin-10
IND	Investigational new drug
LBP	Live biotherapeutic product
LC-MS/MS	Liquid chromatography–tandem mass spectrometry
LTB4	Leukotriene B4
MDRO	Multidrug-resistant organism
NAAT	Nucleic acid amplification test
NF-κB	Nuclear factor kappa B
PGE2	Prostaglandin E2
QC	Quality control
QFM	Quantitative fractionated microbiota
qPCR	Quantitative polymerase chain reaction
rCDI	Recurrent *Clostridioides difficile* infection
ROS	Reactive oxygen species
SAE	Serious adverse event
SCFA	Short-chain fatty acid
SER-109	A defined consortium of purified Bacillota spores (marketed as VOWST)
STAT3	Signal transducer and activator of transcription 3
STEC	Shiga toxin-producing *Escherichia coli*
TEAE	Treatment-emergent adverse event
TNF	Tumour necrosis factor
Treg	Regulatory T cell
UC	Ulcerative colitis
WMT	Washed microbiota transplantation
